# The New Standard Is Biodigital: Durable and Elastic 3D-Printed Biodigital Clay Bricks

**DOI:** 10.3390/biomimetics7040159

**Published:** 2022-10-10

**Authors:** Alberto T. Estévez, Yomna K. Abdallah

**Affiliations:** 1 iBAG-UIC Barcelona, Institute for Biodigital Architecture & Genetics, Faculty of Architecture, Universitat Internacional de Catalunya, 08017 Barcelona, Spain; 2 Department of Interior Design, Faculty of Applied Arts, Helwan University, Cairo 11111, Egypt

**Keywords:** biodigital, elastic clay bricks, 3D printing, controlled material deposition, mechanical properties, compressive strength, sustainability

## Abstract

In a previously published study, the authors explained the formal design efficiency of the 3D-printed biodigital clay bricks 3DPBDCB: a project that aimed to change the conventional methods of clay brick design and mass production. This was achieved by employing the behavioural algorithms of reaction-diffusion and the shortest path that were extracted from the exact material physical properties and hydrophilic behaviours of clay and controlled material deposition 3D printing to create sustainable clay bricks. Sustainability in their use in the built environment and their production processes, with full potential sustainability aspects such as passive cooling, thermal and acoustical insulation, and bio receptivity. The current work studies the mechanical properties of the 3D-printed biodigital clay bricks as elastic and durable clay bricks whose properties depend mainly on their geometrical composition and form. Each of the three families of the 3D-printed biodigital clay bricks (V1, V2, V3) includes the linear model of a double line of 0.5 cm thickness and a bulk model of 55% density were tested for compression and elasticity and compared to models of standard industrial clay bricks. The results revealed that the best elasticity pre-cracking was achieved by the V2 linear model, followed by the V3 linear model, which also achieved the highest post-cracking elasticity—enduring until 150 kN pre-cracking and 200 kN post-cracking, which makes the V3 linear model eligible for potential application in earthquake-resistant buildings. While the same model V3-linear achieved the second-best compressive strength enduring until 170 kN. The best compressive strength was recorded by the V1 linear and bulk model enduring up to 240 kN without collapsing, exceeding the strength and resistance of the industrial clay bricks with both models, where the bulk and the perforated collapsed at 200 kN and 140 kN, respectively. Thus, the mass production and integration of the V1 bulk and linear model and the V3 linear model are recommended for the construction industry and the architectural built environment for their multi-aspect sustainability and enhanced mechanical properties.

## 1. Introduction

Testing an object’s structural performance and mechanical properties always depends on the interplay of a material’s chemical and physical composition and the shape/form that employs this material to translate its geometrical nature into physical reality and how the surrounding forces affect this intimate relationship between material and form. It is fair to say that this equilibrium between material and form has existed forever in nature by the physiological (physical/chemical performance, anatomical (geometrical composition), and histological (material composition) agreement for the survival of one living/or non-living creature. Thus, every creature in nature is 100% efficient in its ideal conditions to deliver its functions and maintain its survivability with no excess materials, either inner or outer (resources), due to its formal coherency and perfect alignment with its materials composition and its formal grammar. When it comes to human creations, on the other hand, this question has been revisited many times since the ancient Egyptian statues that were carved in granite, basalt, and hard coherent stones with settled body postures that have almost no flying parts or hanging angles or bridges due to the hardness of the material to sculpt, and thanks to this combination of hard material and conservative forms, most of these ancient Egyptian statues did not suffer breakages or missing parts for 7000 years, e.g., the Statue of the Goddess Sekhmet curved in basalt ca. 1390–1352 BC [1,2], and the statue of The Menkaure Triad carved in Grey-green Schist from the Old Kingdom, Dynasty 4, Reign of Menkaure (ca. 2494–2472 BC) that preserved perfectly all of its parts without even a single scratch [3,4], which was not the case of the free-form Greek statues that used marble [5] which is less coherent than hard stones as granite and basalt, although easier to sculpt. These romantic postured Greek figures, e.g., the statue of *Venus de Milo* carved in marble between 150 and 125 BC, had both of its arms broken [6]. The statue of *Aphrodite of Knidos* was also carved in marble by Praxiteles in the fourth century BC. An arm and a leg were broken from the statue’s left side [7]. With their perfectly sculpted realistic details, these statues did not preserve many of their parts over time due to their flying parts (arms, wings, etc.). This was also due to marble’s softness in comparison to other stones, such as basalt [8]. Comparing these two examples from a material consumption point of view: the Ancient Egyptian statues consumed more material, but it was all utilised in the actual form of the resulting sculpture being bulky with fewer voids. However, although Greek statues consumed less material in the actual final form of the statue, a nearly equal amount of material was wasted during the carving of one statue. Furthermore, from a fabrication sustainability point of view, carving marble is easier than carving basalt. Nevertheless, the conservative Egyptian forms and postures of their statues reduced the workload in comparison to the Greek statues’ flowing postures. Thus, it is obvious that back then, a designer/sculptor/builder had to give away either the free-form for the durability and customised consumption of the material or vice versa, since the tools then were a chisel and a hammer, and the material was a block of stone. Today, there is no excuse, the material, the tools, the technology, and the form are all liberated by digitalisation and complete control. Now it is possible to design digitally free forms and use controlled material deposition to allocate the material exactly where needed without wasting any while achieving an enhanced structural performance with this combination. Our role as architects, engineers, and designers is to validate the durability of this combination: our forms realised in our sustainably managed materials.

Recently, it would be fair to say that 3D printing is shaping the future of the construction industry. We have witnessed multiple scaling-up architectural 3D-printed projects [9,10,11]; in various materials, including concrete [12,13], clay-based [14,15], steel [16,17], and more and on an international scale. The only obstacle to widescale adoption of this technology is the advanced equipment required for architectural-scale printing and its related cost. Thus, as proposed earlier in the first part of the article on the 3D-printed biodigital clay bricks [14], the key is pixelating the mass and decentralising the industry through 3D printing of clay brick following the standard dimensions of industrial brick, while minimising the material consumption through its optimised formal design and controlled material deposition. In this case, the clay brick is of a cheap and affordable material (clay), with affordable printing technology (to 3D print a volume of a standard brick) that anyone can use and afford, providing minimum material consumption and a physiological structurally optimised form.

**Figure 1:**
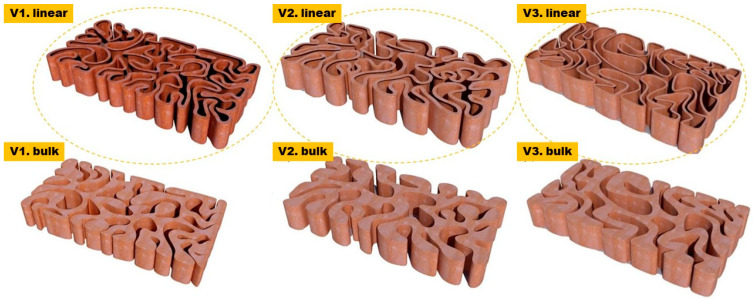
Exhibits the three design families V1, V2 and V3 of the biodigital clay bricks; each design family includes linear and bulk models. The two examples exhibited in the figure of each design family show the 0.55 thickness linear brick model and the 55% density bulk brick model that are tested for their mechanical properties in the current study.

Thus, in the current work, the mechanical properties of the 3D-printed biodigital clay bricks are tested and focus on two main questions: elasticity of the form (to prove its futural potential for earthquake resistance) and durability of the form (to prove its application as a structural element in the architectural built environment, not just as a cladding). The three families of 3DPBDCB: V1, V2, and V3, exhibited in Figure 1, were tested to answer these two questions. Each family, including one linear (hollow model) of a double line of 0.5-line thickness and one model of 55% density bulk bricks, were tested for elasticity and compression, which corresponds to the elasticity and coherence of their geometrical forms since they are all made from the same material, clay. Mainly highlighting the mechanical properties of the form rather than the material to prove that less material could provide enhanced mechanical properties if it used the correct form logic that is generated from the material behaviour itself. As these forms of the three families V1, V2, and V3 were generated following a form-finding process, where a reaction–diffusion model was created upon the simulation of water/liquid diffusion in a standard dimension cuboid clay brick to generate the path of this diffusion pattern through the clay brick volume. Furthermore, a shortest path algorithm was employed to connect the resulting latest points to absorb liquid in the space of the clay brick volume with the minimum distance between every two points to guarantee their structural efficiency minimising the span between every two main points in the resulting diagram from the reaction–diffusion simulation [14]. The various design families result from the third design step, which was to examine these resulting shortest path forms for the final brick resistance to cracking by applying a standard load simulation in Kangaroo, resulting in different design iterations: V1, V2, and V3. The optimum design iterations for structural behaviour were selected to be further tested for various material densities, varying the density of each brick in a bulk brick model from 25% to 55%, 75%, and 95%, and a linear or hollow brick model starting from the thickness of 0.25 cm to 0.55 cm, 0.75 cm, and 0.95 cm for each iteration (V1, V2, and V3). The three design families are exhibited in Figure 1.

## 2. Materials and Methods

This section exhibits the experimental procedures to test the main hypothesis of this project and study that the geometrical design alone can enhance the mechanical properties of clay brick in terms of elasticity and durability. To prove this, the material of all bricks is unified and prepared under the same conditions. The 3D printing technique and machines were unified for 3D printing of all the bricks, and the firing was done under unified conditions for all bricks as well. The mechanical tests in this study were conducted under unified conditions to prove without a doubt that the resultant performance of each brick is only due to its geometrical design and physiological efficiency of its function. Unifying the use of one material for printing all the bricks neutralises the effect of the mechanical properties of the material on the overall structural performance of each brick, which is a pivotal point in the study that controls the experimental methods and the analysis of the results, as exhibited later. It is also important to point out that the aim of the current study is to identify the mechanical properties of each geometrical design of the 3DPBDCB group in comparison to the conventional industrial popular clay bricks, both bulky and hollow, to investigate the scaling up of this project “3D Printed Biodigital Clay Bricks” for mass production and real application in the architectural construction industry. This will prove the overall aim of the project to shift to 3D printing clay bricks as a sustainable and decentralised mass production method that will change the economy and the workflow in the construction industry.

### 2.1. 3D Printed Biodigital Clay Brick Production

**Figure 2:**
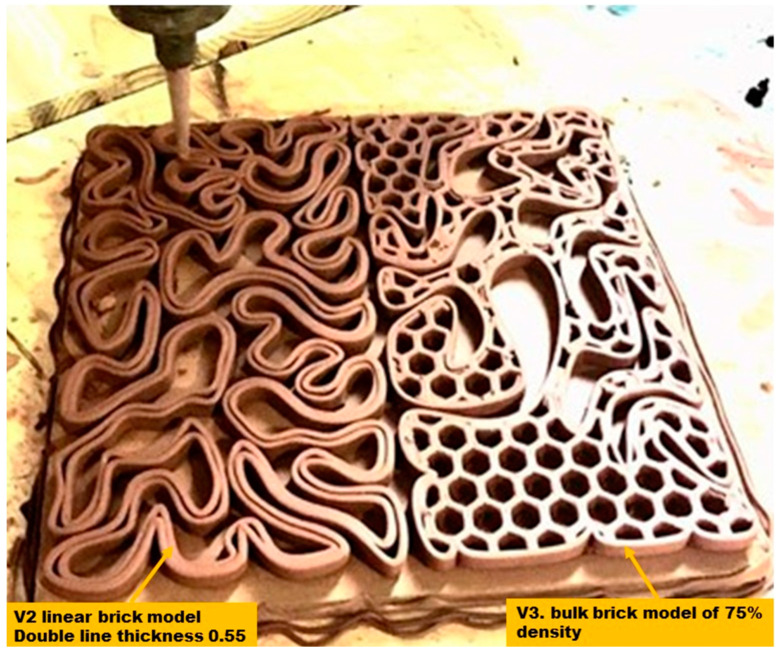
The 3D printing process of the 3D-printed biodigital clay bricks exhibiting the differentiation in printing settings between the linear model (V2) of double line each of 0.55 cm thickness and a bulk model (V3) with honeycomb infill. 3D printing managed by La Maquina by Noumena. (Photo by authors).

The biodigital clay bricks were digitally designed using Rhinoceros Grasshopper and supportive plugins as described in [14]. The digital design was prepared for 3D printing with a specific clay type purchased and prepared following the supplier’s instructions (https://www.angelacolls.com/en/pf-3d/product/5641. Accessed on 6 August 2022). This clay is a traditional red earthenware, adapted for 3D printing, being a polyvalent clay with enhanced plasticity. Its specifications, according to the manufacturer, are as follows: firing range: 970–1055 °C, water content (throwing): 25%, plasticity (IP Atterberg): 17, calcium carbonate content (CaCO_3_): 10%, drying shrinkage: ≈7%, firing shrinkage (1000 °C): 0.6%, porosity (water absorption at 1000 °C): 13.5%, dry bending strength: 6.6 kN/mm^2^, fired bending strength (1000 °C): 27.9 kN/mm^2^, and thermal coefficient (25–500 °C): 77.7 × 10^−7^ °C^−1^. The material preparation and 3D printing process were managed by Noumena/La Maquina (https://noumena.io/en/; https://lamaquina.io/en/. Accessed on 9 October 2022). The design files were prepared for printing using the software: SIMPLIFY 3D, to be printed with the DELTA WASP 3MT FAB 3D printer with a nozzle size of 4 mm. The printing infill pattern of the bulk models was adjusted to honeycomb with 1 cm diameter, while the linear models’ line thickness was adjusted to double line each of 0.5 cm, as exhibited in Figure 2. The printing and firing time for the 12 bricks was four days, using only one clay printing machine, printing just two bricks at a time. Moreover, taking into consideration the required time for all 12 bricks to bone-dry before firing, which in standard bricks takes between 12 h and 7 days per piece before starting the firing process to avoid an explosion inside the kiln [18]. Thus, the production time reached in the current study is competitive in regards to being a pilot scale experimental research project conducted with the minimum available equipment and not using mass production digitalised/robotised production lines in factories [19], taking into consideration that the current study used only one clay-3D printer and a minimum drying time of 12 h.

For the mechanical tests, only two models of each family (V1, V2, V3) were selected based on balancing the material amount used in their 3D printing with their structural coherency (resistance to cracking post-firing). These were a linear model of double line each of 0.5 cm thickness and a bulk model of 55% density with an infill pattern of the honeycomb (diameter 1 cm).

All the tests were carried out at the facilities of the CATMech-Litem research group, placed at C/Colom, 11. 08222 Terrassa (ESEIAAT School, UPC). The tests were conducted under the supervision of the group director: Professor Lluís Gil., and with the kind help of Professors Ernest Bernat, Luís Mercedes, and Andrea Avellaneda.

### 2.2. Elasticity Test

This test aimed to prove the elasticity/flexibility of the 3D-printed biodigital clay bricks to demonstrate their potential use as structural bricks that can resist earthquakes and exhibit flexibility both before and after cracking and deformation. For conducting this test only, the linear models of each family of the biodigital clay bricks were tested (V1, V2, V3) due to their higher resistance to cracking post-firing and since they are also the models that consumed less material in their 3D printing process in comparison to the bulk models of their typical families respectively. The bulk models of each family exhibited less potential for elasticity and more tendency to crack post-firing. Thus, they were not tested for elasticity but only for compression, unlike the linear models that were tested for both elasticity and compression.

**Figure 3:**
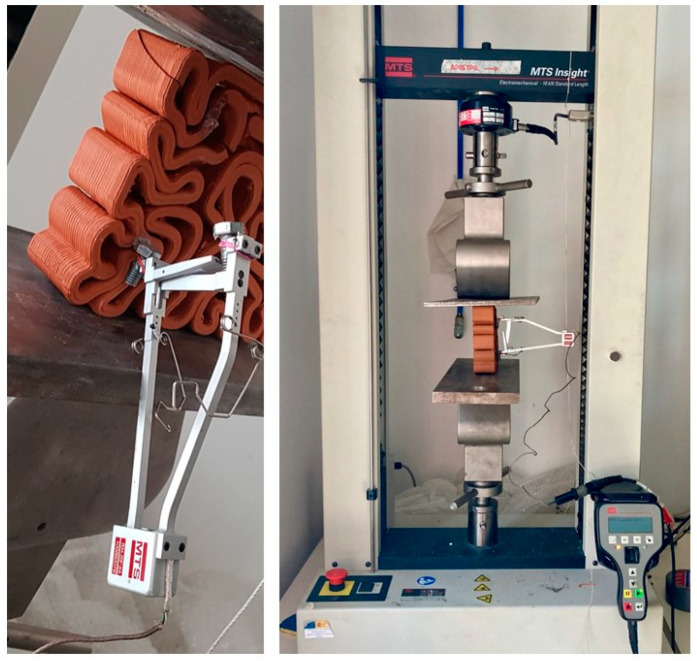
Showing the device used to apply the elasticity test of the 3DPBDCB, using the MTS Insight 10 kN electromechanical press. At the facilities of the CATMech-Litem research group (ESEIAAT School, UPC). (By authors).

The deformability tests aimed to measure the strain of the 3D-printed biodigital clay bricks were carried out in the MTS Insight 10 kN electromechanical press. The load was applied by displacement control at 1 mm/min. Force and displacement of the crosshead were simultaneously recorded at 2 Hz. An external extensometer was installed onto the lateral face of the sample to measure the strains due to the applied loads, as exhibited in Figure 3. Extensometer measurements were also recorded at 2 Hz.

### 2.3. Compression Test

**Figure 4:**
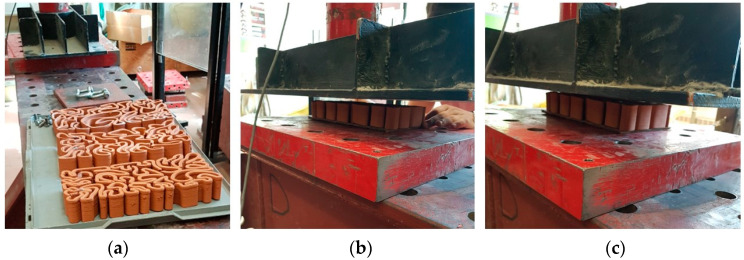
Compression Test. exhibiting the compression test for the 3D-printed biodigital clay bricks conducted by an oleo hydraulic actuator MTS of 250 kN range, and force was applied at 1 kN/s: (**a**) the linear models of (V1, V2, V3) families of the 3DPBDCB, (**b**,**c**) shows the adjustment of the bricks under the actuator. At the facilities of the CATMech-Litem research group (ESEIAAT School, UPC). (By authors).

For testing the durability of the 3DPBDCB, both bulk and linear models of each family (V1, V2, V3) were tested for their resistance to compression. The compression tests were conducted using an oleo hydraulic actuator MTS of 250 kN range. Force was applied at 1 kN/s. Force and displacement were recorded at 5 Hz. Two cardboard plates were placed at both contact surfaces of the bricks with the rigid steel tooling. These were installed to avoid local stress concentrations. Figure 4 exhibits the process of the compression test for the 3DPBDCB.

## 3. Results and Discussion

Since the main research question in the current study is to prove the structural efficiency of the form; the geometry that was generated by the form-finding process based on the clay material physical behaviour of reaction–diffusion and shortest path algorithms, it is important to point out that the current study does not aim to prove the structural efficiency of the material (clay), but rather the form. Therefore, the clay material was neutralised in the current study for 3D printing all the biodigital clay bricks design families (V1, V2, V3) with their linear and bulk models. This, in turn, neutralises everything to do with the material’s mechanical properties, of its Young’s modulus and compression values and highlights the overall performance of each of the 3D-printed biodigital clay bricks.

The second key point in the analysis of the results in this study is the nonuniform design of each of the 3D-printed biodigital clay bricks design families with both linear and bulk models. In turn, this excludes their from following the conventional methods of estimating their mechanical properties since there is no one uniform cross-section of each brick or even multiple cross-sections due to the continuous formal transition of each part in each of the 3DPBDCB. These lattice-like bricks are not even following a uniform grid but rather a digitally generated morphogenic form that is totally resulting from the reaction–diffusion–shortest path diagrams that are the result of the physical simulation of clay hydrophilic properties. Thus, to understand the mechanical performance of each brick of the 3DPBDCB, the overall performance of the entire brick will be exhibited and discussed, away from the inclusion of material properties and uniform estimations.

### 3.1. Material/Fabrication Conditions Standardisation

This study aims to prove the geometrical design and form structural efficiency of the 3D-printed biodigital clay bricks, and the material composition, preparation and printing were unified in all the 3DPBDCB to neutralise the effect of the material´s own mechanical properties. However, the authors are aware of the differential role of the material in the overall structural performance of the bricks. The clay material was chosen for the 3D printing process of this project due to its availability, cost-effectiveness, ease in preparation and 3D printing, as well as its climatic and sustainable merits, being useful in thermal insulation and passive cooling, and recyclable. All these characteristics of clay have made it among the first choice in many recent 3D printing architectural projects, along with concrete and other adobe-based materials [20,21,22]. Most of the clay building materials include illite along with quartz, feldspar, carbonates and iron oxides [23,24,25,26]. Preparation of illite-based ceramics is based on firing the ceramic bodies at temperatures from 700 °C to 1100 °C, which solidifies the ceramic body and enables its sufficient mechanical strength. On a microstructural scale, the porosity of the ceramic body is significantly decreased partially during the solid-state sintering at temperatures below 850 °C and mainly during the liquid-state sintering at temperatures above 900 °C [23,26]. Furthermore, these illitic clays are known for good vitrification, with the glassy phase part in the fired body reaching over 50% mass. These characteristics, in turn, affect the structural performance of ceramics under strain and stress. To quantify these characteristics of a material, Young’s modulus (*E*) is an important indicator of the mechanical characteristics of ceramics, as it depends on external influences and intrinsic properties of the studied material. Thus, Young’s modulus enables the indirect study of the microstructure of ceramics giving insight into the effects of the preparation technology (mineral composition, forming, drying, firing) on ceramic structural performance [27,28]. Young’s modulus, or the modulus of elasticity in tension or compression, is a mechanical property that measures the tensile or compressive stiffness of a solid material when the force is applied perpendicular or lengthwise. It quantifies the relationship between tensile or compressive stress (force per unit area) and axial strain (proportional deformation) in the linear elastic region of a material and is determined using the formula [29].{\displaystyle E\equiv {\frac {\sigma (\varepsilon)}{\varepsilon }}={\frac {F/A}{\Delta L/L_{0}}}={\frac {FL_{0}}{A\,\Delta L}}}.

$$E=\frac{\sigma \left(\varepsilon \right)}{\varepsilon }=\frac{F/A}{\Delta L/L0}=\frac{\mathrm{FL}0}{A\Delta L}$$

where **{\displaystyle E}E** is Young’s modulus (modulus of elasticity), **{\displaystyle F}F** in Newton is the force exerted on an object under compression or tension, **{\displaystyle A}A** in m^2^ is the actual cross-sectional area, which equals the area of the cross-section perpendicular to the applied force. The ratio of F/A is identified as stress, **{\displaystyle \Delta L}▲L** (unitless quantity); the strain is the amount by which the length of the object changes ({\displaystyle \Delta L}positive if the material is stretched, which is called *tensile* strain, and negative when the material is compressed and is called *compressive*), and **{\displaystyle L_{0}}L_0_** is the original length of the object.

At near-zero stress and strain, the stress–strain curve is linear, and Hooke’s law describes the relationship between stress and strain, which states that stress is proportional to strain in linear isotropic materials. The coefficient of proportionality is Young’s modulus. The higher the modulus, the stiffer the material and the more stress is needed to create the same amount of strain. This, in turn, describes the material strength by calculating the maximum stress a material can withstand while staying in elastic-reversible deformation. *Geometric stiffness describes a global characteristic of the body that depends on its shape and not just on the local properties of the material*. For example, an I-beam has a higher bending stiffness than a rod of the same material for a given mass per length. *This geometric stiffness is the focus of the current study since the material used is unified for the 3D printing of all the bricks, which enables the clear identification of the effect of geometric design on the mechanical and structural performance of the 3DPBDCB*.

**Figure 5:**
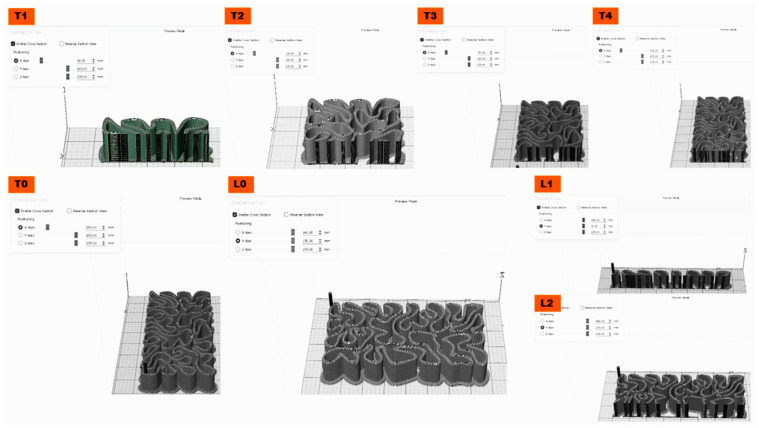
Shows one linear brick model of the V2 family of the 3D-printed biodigital clay bricks showing the nonuniform cross sections along both axes (longitudinal and transversal) and consequently the numerous collective cross sections that each brick of the biodigital bricks has. T0 and L0 represent the two axial views of the V2 linear model full brick (T: transversal, L: longitudinal); from T1 to T4 shows the various transversal cross sections of the V2 linear model brick that were captured every 50 mm along the axis; L1 and L2 are the two longitudinal sections captured every 50 mm along the *Y*-axis—showing the nonuniform morphogenic forms of the biodigital clay bricks.

It is known that Young’s modulus is not always the same in all orientations of a material. However, ceramics are considered an isotropic material and their mechanical properties are the same in all orientations [30], with bulk modulus ranging from 6 and 12 GPa [31] and Young modulus of 0.2~4 MPa [32,33]. However, the material role in the strength and elasticity of the 3DPBDCB is not the question here. It is the geometric design of the 3DPBDCB with its nonuniform and morphogenic design that determines the isotropic property and the overall mechanical properties of these bricks. In this case, Young’s modulus will not change depending on the direction of the force vector at each point but according to the cross-sectional area or, more accurately, the cross-sectional areas since there are numerous profiles of each brick of the 3DPBDCB with numerous cross sections at each section of the brick as exhibited in Figure 5. Furthermore, even the accurate calculation of the sum of cross-sectional areas of each brick of the 3D-printed biodigital clay bricks will not give a real understanding of the brick’s overall mechanical and structural performance since the sectioning axis will produce various cross-sections along the length of the brick as exhibited in Figure 5, *as well as the uncertainty of the linear isotropic nature of the form. This highlights a very important aspect of isotropic or anisotropic geometrical designs, which is part of the scientific question in the current study*. *Thus, even the superposition principle derived from Hooke’s law addressing morphogenic or shape-changing structures would not be eligible for application since the 3DPBDCB can’t be considered linear or non-linear systems before testing their mechanical properties in a holistic way*. *Thus, in the current study, the overall performance of the 3DPBDCB under various force values and how much they would endure will be an insightful measurement of their efficiency*.

### 3.2. Elastic 3D Printed Biodigital Clay Bricks

According to Griffith’s theory, Young´s modulus is directly proportional to mechanical strength [34,35]. A solid material will undergo elastic deformation when a load is applied to it in compression or extension. This elastic deformation is reversible, meaning the material returns to its original shape after removing the load.

In the current study, the main goal is to test the geometrical design effect on the elasticity and strength of the 3DPBDCB by excluding the effect of the material’s properties- which was discussed previously in the literature reporting the elastic behaviour of clays [36,37,38,39] and elsewhere—in addition, analysing the results considering the holistic mechanical and structural performance of the entire brick as justified in the previous section.

**Figure 6:**
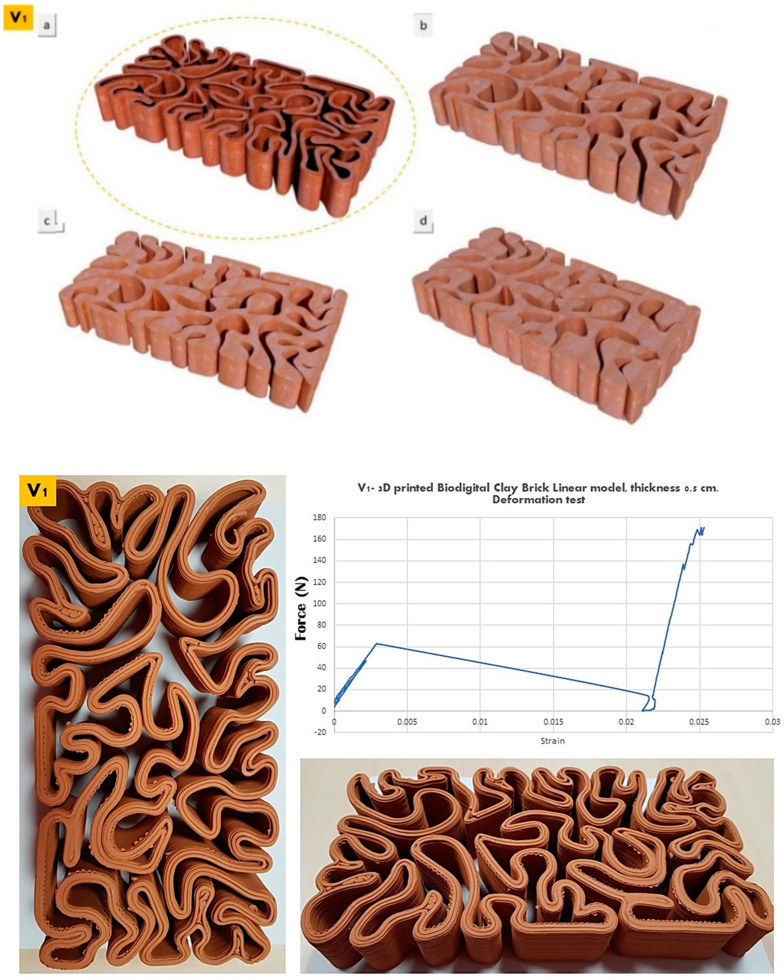
Exhibits the linear brick model of the V1 family of the biodigital clay bricks with double line linear thickness of 0.5 cm in 3D renders (**top**) as (**a**) id the tested linear brick model while (**b**–**d**) are the bulk models of the same family with variant densities. and in physical 3D-printed model (**bottom**). The graph exhibits the results of the deformation test for this brick, showing the relation between force (N) (*Y*-axis) and strain (*X*-axis). By excluding the nonuniform cross-section area, the relation between F and ▲L gives an indicator of the overall elastic behaviour of the brick. The V1 linear model achieved its highest strain under 60 kN with strain of 0.0025 pre-cracking and significantly increased elasticity post-cracking, reaching 0.025 strain under 170 kN.

**Figure 7:**
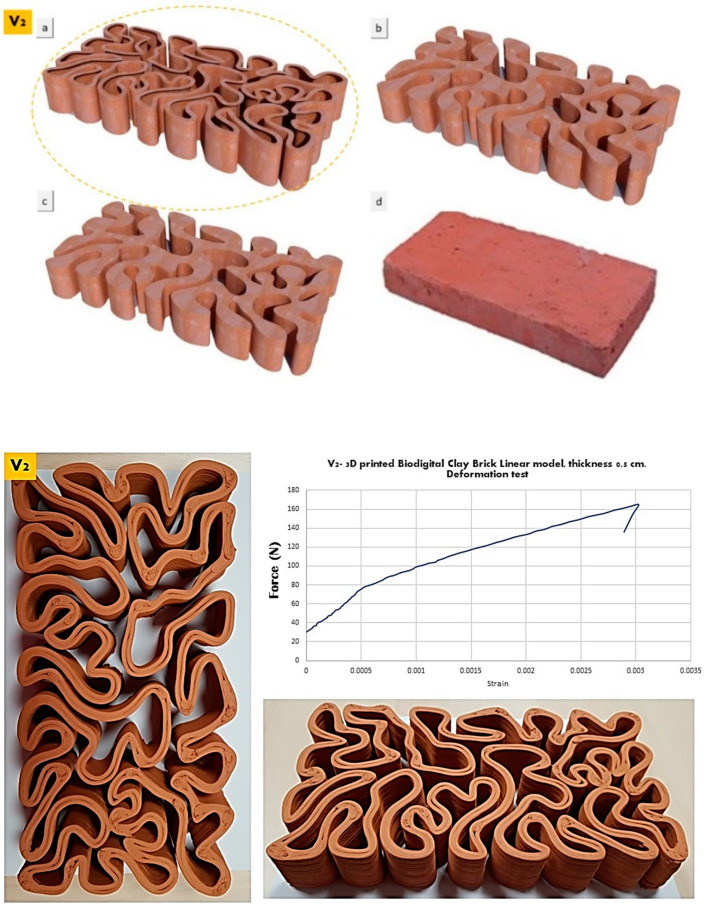
Exhibits the linear brick model of the V2 family of the biodigital clay bricks with double line linear thickness of 0.5 cm in 3D renders (**top**) as (**a**) is the tested linear brick model while (**b**–**d**) are the bulk models of the same family with variant densities. and in physical 3D-printed model (**bottom**). The graph exhibits the results of the deformation test for this brick, showing the relation between force (N) (*Y*-axis) and strain (*X*-axis). The V2 linear model biodigital brick exhibited its highest strain (pre-cracking) of 0.003 under 160 kN, with continuously decreased elasticity post-cracking.

**Figure 8:**
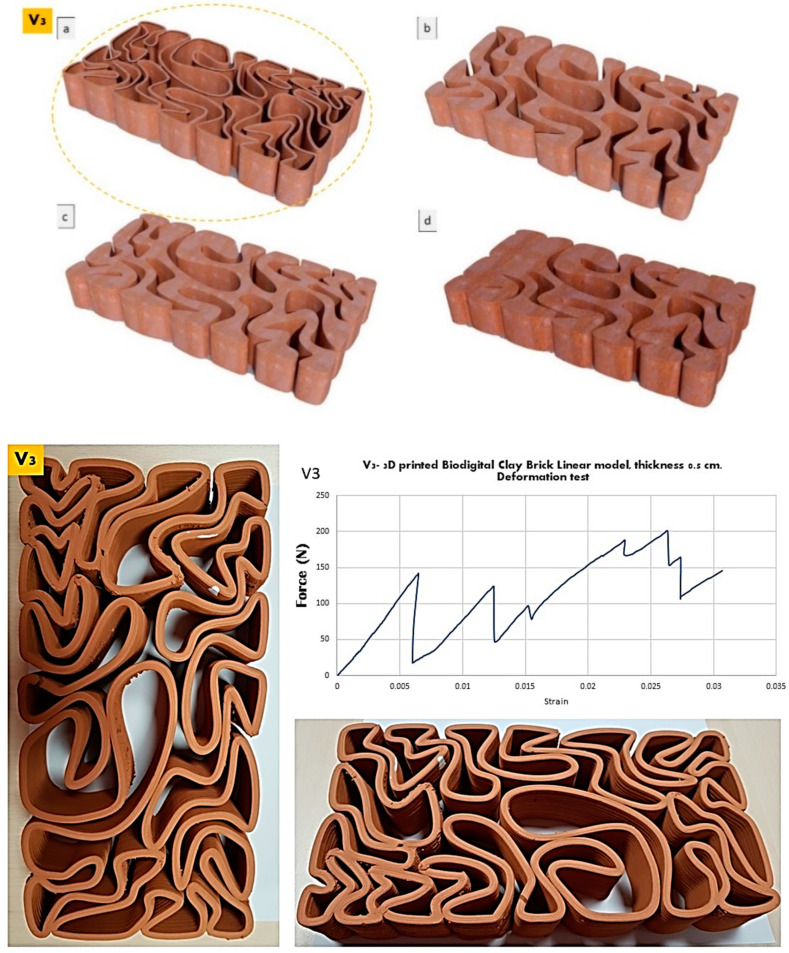
Exhibits the linear brick model of the V3 family of the biodigital clay bricks with double line linear thickness of 0.5 cm in 3D renders (**top**) as (**a**) is the tested linear brick model while (**b**–**d**) are the bulk models of the same family with variant densities. and in physical 3D-printed model (**bottom**). The graph exhibits the results of the deformation test for this brick, showing the relation between force (N) (*Y*-axis) and strain (*X*-axis). The V3 linear model exhibited the highest strain of 0.005 under 150 kN pre-cracking and the highest strain of 0.026 under 200 kN post-cracking. This was the best elasticity among all three linear brick models of the biodigital clay bricks.

The elasticity test results depicted in Figure 6, Figure 7 and Figure 8 exhibit the performance of each of the linear models of the 3D-printed Biodigital Clay Brick families (V1, V2, V3), respectively.

The linear brick model of the V1 family recorded the least elasticity pre-cracking, as it started cracking under 60 kN with a strain of 0.0025 and continued to decrease its strain to 0.022 under nearly 10 kN, as exhibited in Figure 6. However, its elasticity increased steadily and significantly until reaching 0.025 strain under ≥170 kN post-cracking. This is justified by the minimum interstitial void spaces between the dense distribution of the brick form curves that are tight with minimum spatial expansion and acute angles. This reduces the void spatial margins that allow expansion and reversible deformation. Thus, this brick elastic performance enhanced significantly post-cracking that allowed the creation of escape or open points for the applied force to go through, which alleviated its effect on the parts of the brick in force transfer from a part or a point to the following. In addition, due to the nonuniform morphogenic form of the brick, the transfer of the force can only be detected by the overall performance of the brick. Therefore, the overall elasticity of the V1 linear model brick enhanced significantly when its coherence was reduced by the effect of cracking.

A higher strain (pre-cracking) was recorded by the V2 linear model brick that exhibited elasticity until 0.003 strain under ≥160 kN before it started to crack, as exhibited in Figure 7, while post-deformation, its elasticity started to decrease steadily. This is justified by the almost equal distribution of solid and void volumes in the space of the brick model, enabling spatial margins for the reversible deformation of its parts, along with the moderate and gradual curvature bending of its lines with wide angles and limited curve length. While due to the same reason of the nearly equal distribution of solid and void, the brick failed to exhibit any elasticity post-cracking due to the significantly decreased coherence of the cracked parts of the V2 brick that affected its overall performance.

The V3 linear model brick achieved the best elasticity and recorded 0.005 strain under 150 kN before cracking and steadily increased its elasticity with fluctuations post-cracking until 0.025 strain under 200 kN. More interestingly, when analysing the graph exhibited in Figure 8, the brick exhibits a near similar elasticity under ≈150 kN at 0.005 strain pre-cracking and under 125 kN at 0.010 strain post-cracking. This implies the parallel elasticity behaviour between the different nonuniform parts of the brick. Furthermore, the overall elasticity of the brick was enhanced post-cracking thanks to its geometrical design that enables wider spatial margins for elasticity as the void spaces are dominant in this design. Another reason is the differential form of the V3 brick that resulted in the deferential curves, angles, and extension. This enhanced elasticity post-cracking suggests the possible use of the V3 linear model as a structural brick with mild earthquake resistance.

After calculating the average of cross sections of each brick and applying the elastic modulus formula described in Section 3.1, the deformability results showed an average apparent elastic modulus of 6.3 MPa. However, comparing the results of all three bricks, the strain values for all three bricks (pre-cracking) ranged from 0.0025, 0.003, and 0.005 for V1, V2, and V3, respectively, which is a slight change in ▲L. Furthermore, these values of strain of the two biodigital clay bricks V2 and V3 were recorded at nearly similar ranges of force from 145 kN to 165 kN, while only V1 was recording 0.0025 strain under 60 kN, which is nearly 40% of the force applied to the other two Bricks V2, V3 before they reached the first breaking point (cracking). This implies that V2 recorded the best behaviour pre-cracking until 160 kN before reaching its first breaking point. This was followed by V3, which tolerated the strain until 150 kN before starting to crack.

While post-deformation V3 recorded the best behaviour by exhibiting increased elasticity of 0.026 under 200 kN post-deformation and showing a parallel graph between post-deformation to the pre-deformation, which implies an almost uniform behaviour of the different nonuniform morphogenic curves of the brick that proves the sufficiency of the geometrical design of this specific brick, furthermore, at each cracking point, the brick’s elasticity increases, making it suitable for potential structural applications. V1 comes in second place for post-deformation elasticity. However, it had a more non-linear behaviour, as the brick exhibited a decrease in elasticity after the first breaking point when it reached under 60 kN until reaching 0 strain under 40 kN when it started to increase its elasticity significantly, reaching 0.025 under 170 kN which is almost a similar strain value achieved by V3 under higher force value of 200 kN. V2 exhibited a steady decrease in elasticity with no fluctuations post-deformation.

These results imply that V3 exhibited the best overall elastic behaviour among the three biodigital clay brick linear models, V1, V2, and V3.

### 3.3. Durable 3D Printed Biodigital Clay Bricks

Cracking influences Young’s modulus and the mechanical strength of ceramics to a large extent [40]. Thus, to test the efficiency of the 3D-printed biodigital clay bricks in terms of elasticity and durability for future potential structural applications in earthquake-resistant architecture, the compression tests were conducted after the deformation test on the same brick samples (with cracks) in the case of linear brick models, to prove their capacity to endure compression forces even after cracking in comparison to industrial hollow bricks, to simulate an earthquake system (tension and compression) [41,42]. These tests prove that the geometrical design efficiency of clay bricks can be adopted for structural efficiency and earthquake resistance as congruent with [43,44]. On the other hand, since the bulk brick models were not tested for deformation as they did not show any elasticity, the full non-cracked samples were also tested for compression in comparison to an industrial bulk brick to prove the 3DPBDCB competence and potential for structural applications and mass production.

*It is important to mention that the deformation test did not depend on comparing the 3DPBDCB linear models with industrial hollow or bulk bricks since conventional industrial bricks don’t depend on elastic behaviour resulting from the geometrical design of the brick but rather on the material properties of the clay/ceramic itself*. There are no unified criteria of comparison between the various geometrical designs of a brick and their elasticity with the conventional cuboid form of an industrial brick that its mechanical properties test depends more on its material and microstructure, as in [45,46], or its assembly in a full clay/masonry-brick wall [47] or the honeycomb form of a hollow brick. Thus, the authors decided to avoid the confusion between material properties and geometry properties that would occur if the 3DPBDCB linear models were compared to industrial bricks in the elasticity test. However, in the compression test, it was mandatory to compare the behaviour of the 3DPBDCB to the standard industrial brick (hollow and bulk) to prove the 3DPBDCB competence with these standard industrial bricks to reproduce them on a wider scale (mass production).

To calculate the compressive strength of each brick, the following formula was used:

F = P/A, where: F = The compressive strength (MPa), P = Maximum load (or load until failure) to the material (N), A = A cross-sectional area of the material resisting the load (mm^2^) [48]. Where in this case, calculating specific compressive strength was possible since the applied force direction was perpendicular to the same surface area of the 3D-printed biodigital clay brick, which enables the calculation of its cross-section area, unlike the case of elasticity modulus calculation as described before in Section 3.1 and Section 3.2).

**Figure 9:**
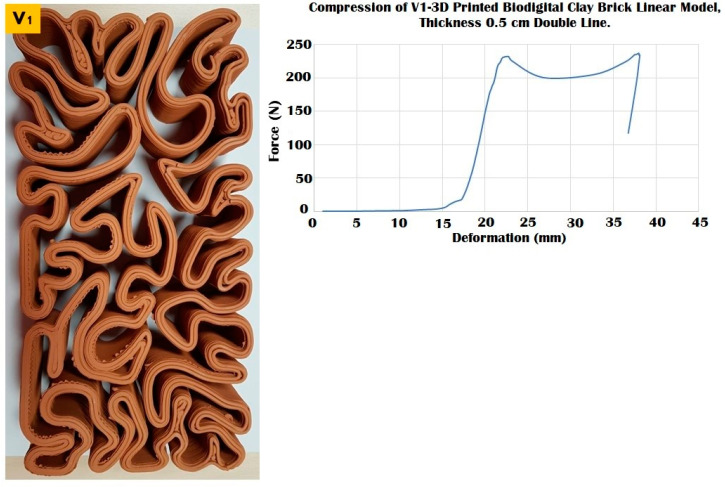
Exhibits the linear brick model of the V1 family of the biodigital clay bricks with double line linear thickness of 0.5 cm in the physical 3D-printed model (**left**). The graph exhibits the results of the compression test for the V1 linear model brick, showing the relation between force (N) (*Y*-axis) and deformation (mm) (*X*-axis). This model exhibited maximum durability by resisting until 240 kN before it started cracking and maintaining its resistance from 25 to 35 mm deformation under 200–250 kN before collapsing at 240 kN.

**Figure 10:**
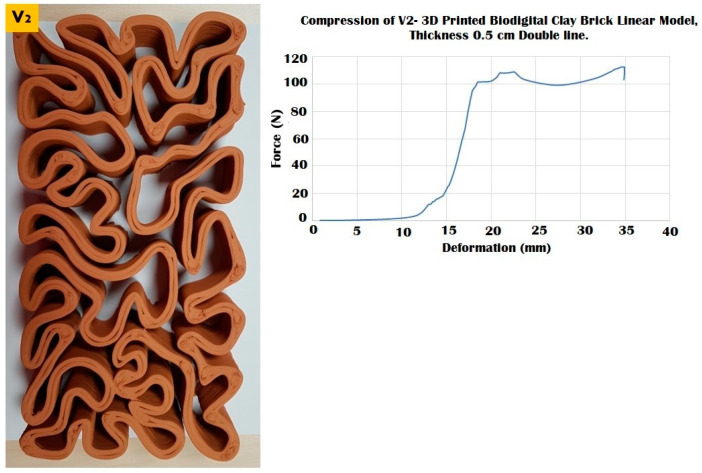
Exhibits the linear brick model of the V2 family of the biodigital clay bricks with double line linear thickness of 0.5 cm in the physical 3D-printed model (**left**). The graph exhibits the results of the compression test for the V2 linear model brick, showing the relation between force (N) (*Y*-axis) and deformation (mm) (*X*-axis). This model exhibited its maximum durability by resisting until 110 kN before cracking and maintaining its resistance from 20 to 35 mm deformation under 100–120 kN before collapsing at 110 kN.

**Figure 11:**
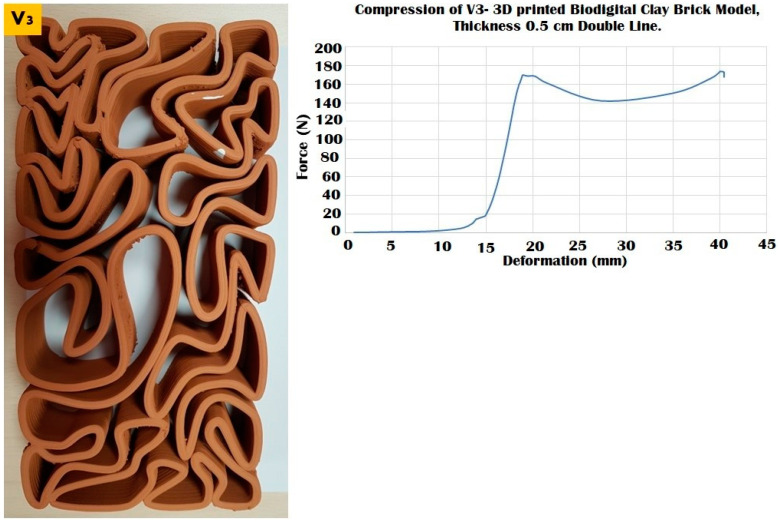
Exhibits the linear brick model of the V3 family of the biodigital clay bricks with double line linear thickness of 0.5 cm in the physical 3D-printed model (**left**). The graph exhibits the results of the compression test for the V3 linear model brick, showing the relation between force (N) (*Y*-axis) and deformation (mm) (*X*-axis) and achieving the maximum durability of this brick, resisting until 170 kN before cracking and maintaining its resistance from 20 to 40 mm deformation under 140–170 kN, before its collapse at 170 kN, which is nearly 77% of the compression-resistance exhibited by the V1 bulk brick model and exceeding the compressive strength of the V1 linear model, while enduring the compressive force effect for a longer time and with higher value of compression reaching 40 mm before it totally collapsed.

The three different linear brick models of V1, V2, and V3 exhibited different resistances to distinct force values ranging from 100 to 250 kN, as exhibited in Figure 9, Figure 10 and Figure 11.

The linear brick model V1 achieved the maximum durability with a surface area of 28,003.6 mm^2^, as exhibited in Figure 9, that tolerated until 240 kN before cracking while maintaining its resistance post-cracking under 200 kN with deformation ranges from 25–35 mm, to gradually increase its resistance again until 240 kN which was its collapsing point at a deformation of 37 mm with an apparent strain of 0.82 and compressive modulus of 0.010.

While the V2 linear brick model with a surface area of 15,980.9 mm^2^ recorded the least resistance among all the three linear models, resisting until 110 kN before it starts cracking and maintaining a slight fluctuating resistance with deformation ranging from 20 to 35 mm under 100–120 kN before its complete collapse under 110 kN at 35 mm with an apparent strain of 0.77 and compressive modulus of 0.007 (Figure 10).

The V3 linear brick model with a surface area of 12,342.9 mm^2^ exhibited a better resistance than the V2 model, as it resisted compression until 170 kN, recording a 20 mm deformation value (Figure 11). After it started to crack, the V3 linear brick model maintained a reduced resistance under 140–170 kN achieving from 25 to 40 mm deformation before it totally collapsed at 0.88 apparent strain and compressive modulus of 0.014. This span of resistance post-cracking exhibited in the amount of deformation pre-collapse, which is higher than the two other linear model biodigital bricks V1 and V2, exhibits the capacity of the geometry of this model V3 to endure compressive force for a longer time and with further deformation values before it totally collapses. *This implies that a combination of the highest resistance of the V1 linear brick model and the longer resistance of V3 could be combined in load-bearing walls for enhanced durability and compression resistance.*

**Figure 12:**
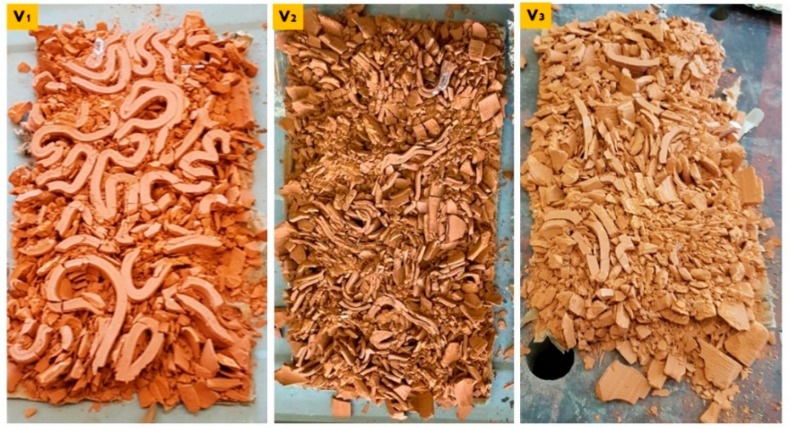
Exhibits comparison between the cumulus of three linear model bricks, V1, V2, and V3, post-collapsing as a result of the compression test. It can be observed that the V1 exhibited the highest resistance since it maintained many of its parts, followed by the V3. At the same time, the V2 recorded the least compression resistance as it was the most affected, as shown in the figure.

These results imply the particular potency of the linear brick model V3 because it achieved the best elasticity performance and the second-best compression resistance performance after the linear brick model V1. As illustrated in Figure 12, the three collapsed bricks V1, V2, and V3 as the result of the compression test. From the cumulus of each brick model, the V1 linear brick model wasn’t even totally ground by the compressive force, followed by the V3, which maintained some of its big pieces without being ground, while the V2 was the most affected by the compression.

**Figure 13:**
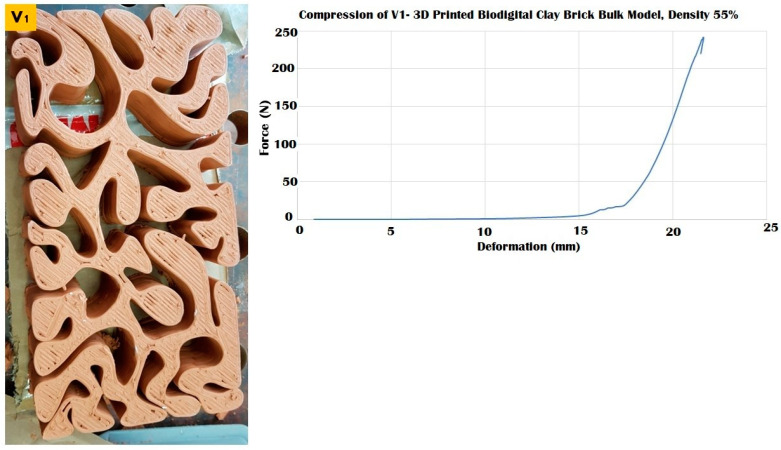
Exhibits the bulk brick model of the V1 family of the biodigital clay bricks with 55% density in the physical 3D-printed model (**left**). The graph exhibits the results of the compression test for this brick, showing the relation between force (N) (*Y*-axis) and deformation (mm) (*X*-axis). The maximum durability of the V1 bulk brick model achieved up to 240 kN before cracking and maintaining its resistance under the same force value without collapsing. This behaviour by the V1 bulk brick model is congruent with its linear model that also resisted until 240 kN before it started to break. The linear model of the V1 collapsed under the same force value after resistance, unlike the bulk model that survived the compression test without collapsing.

The bulk brick model V1, with a surface area of 27,340.14 mm^2^, exhibited the maximum compression resistance as well (Figure 13), resisting until 245 kN when it reached its first breaking point and started to crack at a maximum reached deformation of 21 mm and apparent strain of 0.46 and compressive modulus of 0.018. Unlike all the other linear and bulk models of the 3DPBDCB, even after cracking, the V1 bulk models did not collapse and maintained their durability, as shown later in Figure 16. This indicates the potential use of this brick model for structural purposes and is recommended for mass production.

**Figure 14:**
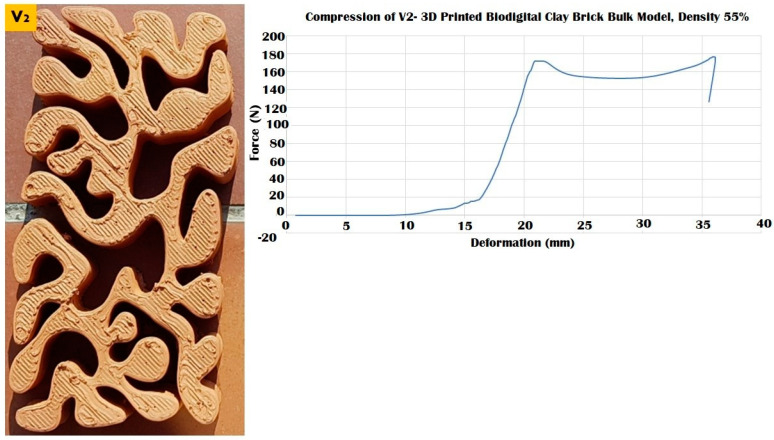
Exhibits the bulk brick model of the V2 family of the biodigital clay bricks with 55% density in the physical 3D-printed model (**left**). The graph exhibits the results of the compression test for this brick, showing the relation between force (N) (*Y*-axis) and deformation (mm) (*X*-axis). The maximum durability achieved of the V2 bulk brick model resisted until 170 kN before cracking and maintaining its resistance under 150–170 kN before collapsing at 36 mm deformation under 170 kN.

The compression resistance of the V2 bulk brick model of 53,014.6 mm2 surface area was better than its linear model resistance. (Figure 14), as it resisted until 170 kN before its first breaking point and maintained resistance at deformation values from 20–35 mm under 150–170 kN before it totally collapsed at 36 mm deformation under 170 kN with an apparent strain of 0.8 and compressive modulus of 0.004. In contrast, the linear model of the V2 only resisted until 110 kN.

**Figure 15:**
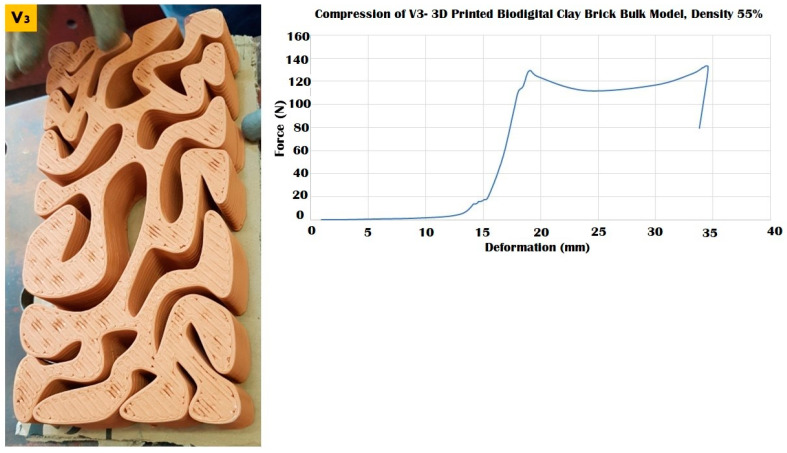
Exhibits the bulk brick model of the V3 family of the biodigital clay bricks with 55% density in the physical 3D-printed model (**left**). The graph exhibits the results of the compression test for this brick, showing the relation between force (N) (*Y*-axis) and deformation (mm) (*X*-axis). The maximum durability was achieved for the V3 bulk brick model at 17 mm under 130 kN and maintained resistance until 34 mm under 135 kN, where it totally collapsed.

Unlike its linear model, the V3 bulk brick model, with a surface area of 28,987.7 mm^2^, came last in compression resistance compared to the V1 and V2 bulk models. It recorded the least resistance to compression and started its first breaking point at 17 mm under 130 kN, despite resisting until 34 mm deformation under nearly the same force value 135 kN, whereafter it totally collapsed (Figure 15), achieving a compressive modulus of 0.007. This was only half the compressive strength when compared to its sister V3 linear brick model that achieved second best resistance to compression collapsing under 170 kN and compressive modulus of (0.014), coming second after the V1 linear brick model that collapsed under 240 kN, which is the highest resistance achieved amongst the linear models. *The V3 bulk model achieved nearly 50% of the resistance exhibited by its sister linear brick model. Which is clear proof that the linear brick model V3 is stronger and more durable than its sister bulk model, proving the hypothesis of the 3D-printed biodigital clay bricks project that, to a great extent, the geometrical design controls the mechanical properties and consequent structural efficiency using less material and controlled material deposition by 3D printing*. Furthermore, this result is supported by comparing the compressive strength of the V1 linear and bulk models, as both resisted until 240 kN. However, the 3DPBDCB geometry doesn’t maintain a linear or uniform mechanical behaviour since the bulk model of the V1 resisted compression better than its sister linear model and survived the test without collapsing. On the other hand, the V3 linear model resisted better than its sister bulk model. The V2 bulk model was better in the compressive test than its sister linear model. From these results, it cannot be claimed that the bulk brick models behave better than their linear sisters in general or vice-versa. Alternatively, *it can surely be concluded that every brick design family is a particular case where its geometry alone affects its mechanical properties, proving the scientific question of the current study*.

**Figure 16:**
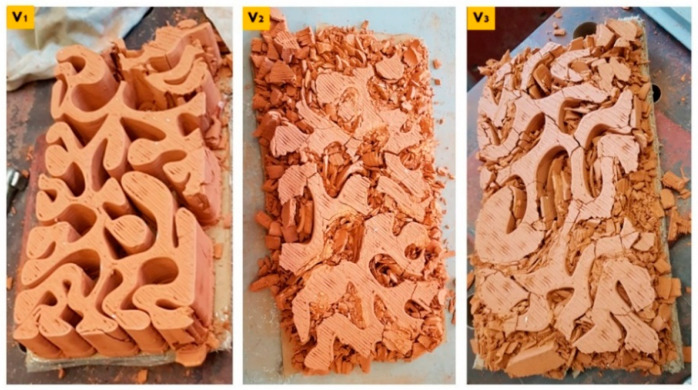
Exhibits comparison between the cumulus of three bulk brick models, V1, V2, and V3, post-collapsing as a result of the compression test. It can be observed that the V1 exhibited the highest resistance since it did not collapse and maintained its coherency all over its body with minimum damage at the peripheries. Followed by V3 despite collapsing at the lowest force value at 135 kN. While the V2 bulk brick model was the most affected.

Comparing the cumulus of the three bulk brick models V1, V2, and V3 (Figure 16). The V1 bulk brick model survived the compressive force until ≥240 kN and only suffered some peripheral breaking. The main body of the brick maintained its coherence without cracking. Followed by the V3 bulk brick model that, despite collapsing under 135 kN, was the least recorded resistance compared to the V1 and V2 (170 kN). However, the horizontal faces of the brick were less damaged than the V2. This can be referred to as the discrepancy in the force value, where each of the two bricks collapsed. However, the difference in force value was only 35 kN, which makes it more sound to consider that the resistance of the V3 bulk brick model was caused by its geometrical design, which is supported by the results achieved by its sister linear model V3 that came second in resistance after the V1 linear brick model resisting until 170 kN (V3).

### 3.4. Durability vs. Elasticity “The New Standard”: 3D Printed Biodigital Clay Bricks

**Figure 17:**
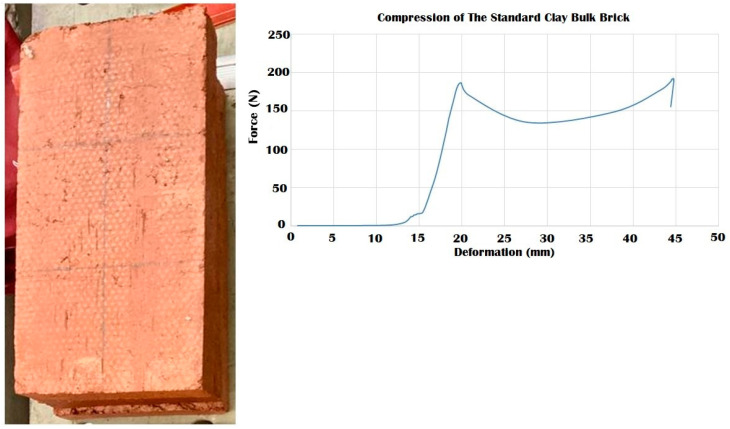
Exhibits the results of the compression test for the standard industrial clay brick. The results show that the maximum compressive force endured by the brick was 190 kN which led the brick to its first cracking point at 20 mm deformation. While the brick maintained resistance from 20 to 44.5 mm deformation under forces ranging from 140–200 kN with a strain value of 0.99, where it finally collapsed at ~200 kN, which is less than the compressive strength achieved by the V1 linear and bulk brick model of the 3DPBDCB that endured until 240 kN before a partial collapse in the case of its linear model and without collapsing in its bulk model.

**Figure 18:**
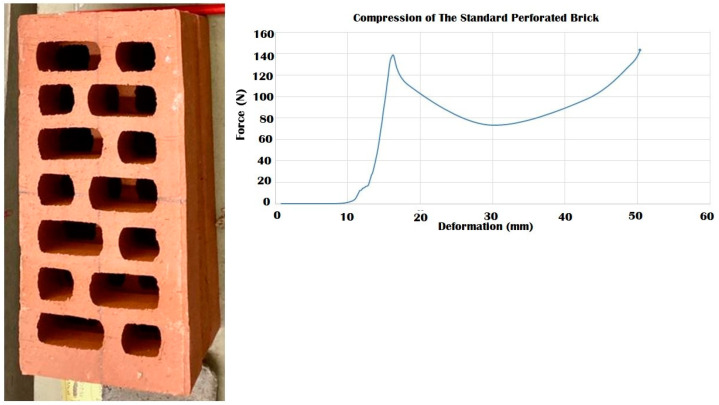
Exhibits the results of the compression test for the standard industrial hollow/perforated clay brick. The results show that the maximum compressive force endured by the brick was 140 kN, which led the brick to its first cracking point at 15.5 mm deformation. While the brick-maintained resistance from 15.5 to 50 mm deformation under forces ranging from 70–140 kN with strain value of 1.11, where it finally collapsed at 50 mm under ~140 kN, which is less than the compressive strength achieved by the V1 linear and bulk brick models. It also recorded less strength than V2 bulk model brick and V3 linear model brick.

To decide on the best mechanical properties and consequent expected structural performance of the 3D biodigital clay bricks, each brick’s behaviour in compression and elasticity should be compared to one another as well as compared to a reference which are two models of industrial clay bricks, one is a bulk standard clay brick model (29.5 × 15 × 4.5 cm), and the other is a hollow brick with the same dimensions. This comparison needed to be ruled by the specific criteria that were proposed in the current study to solve the mechanical performance study of the nonuniform morphogenetic forms of the 3DPBDCB, which did not bend to the conventional method of estimating elastic modulus or compressive strength and that required a holistic approach in evaluating the mechanical properties of each brick. Thus, the standard clay brick (29.5 × 15 × 4.5 cm) with a 44,250 mm^2^ surface area was only examined in the current study for compression (Figure 17). Its hollow model with the same dimensions and a 23,700 mm^2^ surface area was also examined for compression (Figure 18). Their elasticity wasn’t tested as they exhibited no tolerance for reversible deformation. Their Young’s modulus reported in the literature is due to their material properties, not their form. Both models of the industrial clay bricks recorded less strength than the values achieved by the V1 bulk and linear models that tolerated until 240 kN, unlike the bulk industrial clay brick that collapsed under 200 kN with a high strain value of 0.99, and the hollow brick that collapsed under 140 kN which is even less than the force values tolerated by the V2 bulk model and the V3 linear model that collapsed at 170 kN. This proves the project’s hypothesis that geometrical design can achieve structural efficiency even when using the same material with less amount through 3D printing a controlled material deposition. *Thus, the authors propose using the V1 bulk and linear model and the V3 linear model as the new standard in clay brick mass production and application in construction since they have both compressive strength and elasticity that emerges from their geometrical design*. In addition to the multi-sustainability aspects of less material consumption, integrating the latest technology of 3D printing in fabrication, bio receptivity, thermal and acoustical insulation, more will be exhibited in the following publications.

Their compressive strength was compared to the compressive strength of the 3DPBDCB in terms of the maximum force they would endure before collapsing and their strain values. Similarly, the comparison between the 3DPBDCB in elasticity was ruled by the maximum force they would endure before cracking, or in other words, irreversible deformation indicated by their strain values. These results are shown in the following matrix (Table 1).

Comparing their mechanical properties with the standard industrial clay bricks. The matrix exhibits the values of maximum compressive force applied perpendicular to the surface, the apparent strain (deformation/thickness), apparent stress (force/surface area), and the compressive modulus. As well as exhibiting the results of the elasticity test for the 3DBDCB linear models according to the Maximum tolerated force pre-cracking and post-cracking. The results revealed that the best compressive strength, maximum tolerated compressive force, and highest compressive modulus (0.018) were achieved by the V1 bulk model, followed by the V1 linear model with the less compressive modulus (0.010), and the V3 linear model with less tolerated force and second highest compressive modulus of 0.014. These results are congruent with the elasticity test results as well, exhibiting that the V3 linear model had the highest overall elasticity (pre- and post-cracking), followed by the V1 linear model in overall elasticity. The V2 linear model only recorded the highest elasticity pre-cracking with a less compressive modulus (0.007), with half of the compressive modulus achieved by the V3 linear model and 70% of the compressive modulus achieved by the V1 linear model.

Comparing the compressive strength and compressive modulus of the 3D-printed biodigital clay bricks of different models (bulk and linear) from different families V1, V2, and V3 with the standard industrial clay bricks, it is evident that the V1 clay brick with both of its models, the linear and the bulk, have excelled over the standard clay brick with its bulk and perforated models. *Proposing the V1 bricks as the strongest and most durable compared to all the 3D-printed biodigital clay bricks and the standard clay bricks*. Followed by the V2 bulk model and V3 linear models. Both of which have excelled over the standard perforated/hollow brick. *Furthermore, the V3 linear model exhibited the best overall elasticity behaviour pre- and post-cracking, which summons up the comparison in its favour, coming second in durability and first in elasticity*.

Thus, the authors recommend the adoption of the V1 linear and bulk models and V3 linear model of the 3D-printed biodigital clay bricks as the new standard clay bricks for the construction industry, achieving competent mechanical properties depending on their specific geometrical design while minimising the material consumption and integrating 3D printing with accurate material deposition to achieve sustainability and a decentralised construction industry.

## 4. Conclusions

The current study reported the mechanical performance analysis of the 3D-printed biodigital clay bricks to test their competence and possible integration in structural applications in the construction industry. This study comes as a second and complementary part of the first published study on the formal efficiency of the 3DPBDCB to prove that these sustainable clay bricks provide an integrated, sustainable solution with less material consumption and enhanced mechanical properties through controlled material deposition 3D printing. The study analysed the mechanical properties of two models (linear and bulk) of the three families (V1, V2, and V3) of the 3DPBDCB, testing their elasticity and compressive strength based only on their geometric design and not their material. Thus, the material composition, preparation conditions and 3D printing conditions were all unified to ignore the mechanical properties and performance of the 3DPBDCB and only study their geometry. The results revealed that the V3 linear model exhibited the best overall elasticity performance pre- (tolerating 150 kN) and post-cracking (tolerating 200) with the second-highest compressive modulus (0.014). The V2 linear model endured the highest force values pre-cracking (160 kN) while failing to show any elasticity post-cracking. Unlike the V1 linear model, its elasticity behaviour was non-linear as it showed the lowest elasticity pre-cracking (60 kN). However, its tolerance was significantly enhanced post-cracking (170 kN), with the third-best compressive modulus (0.010). The results of the compression test revealed that the V1 linear and bulk models achieved the highest compressive strength exceeding all the bulk and linear models of the 3DPBDCB bricks as well as the standard industrial bulk and perforated clay bricks, especially the V1 bulk model that exhibited the minimum compressive strain value 0.46 and maximum compressive force tolerance until more than 240 kN, which it survived without collapsing, achieving the highest compressive modulus as well (0.018). This was followed by the V3 linear model (170N). The results also revealed congruency between the elasticity and compression results since the best in strength (240 kN), which is the V1 linear model, has the least elasticity pre-cracking (60 kN). While the best in elasticity pre-cracking (160 kN), which is the V2 linear model, is the least in strength (110 kN). The second best in elasticity pre-cracking (150 kN) and best in elasticity post-cracking (200 kN) is the V3 linear model, which achieved the second-best compressive strength (170 kN) as well. Although these results imply a linear and isotropic behaviour of the bricks, comparing their stiffness with their elasticity. However, the differentiation between the V2 linear and bulk models, in compressive strength, as the linear is less strong than the bulk, as well as the flipped differentiation between the V3 linear and bulk models (as the bulk is less strong than the linear), still exhibits non-linear and anisotropic behaviour among the 3DPBDCB bricks and even within the one design family (V2 and V3). Thus, the authors have chosen the V1 linear and bulk models as the most potent for integration in structural applications since they achieved the maximum compressive force, which exceeded the standard industrial clay bricks. The V3 linear model was also chosen as it achieved the second-best strength and best overall elasticity.

## Figures and Tables

**Table 1:** Matrix of mechanical properties of the 3DBDCB bricks resulting from the deformation and compression test.

3DPBDCB Model	Maximum Compressive Force	Apparent Strain (Normal)	Apparent Stress	Compressive Modulus	Elasticity According to Maximum Parallel Force Tolerance.	
		▲L/L_0_	F/A	E	Pre-Cracking	Post-Cracking
V1 Linear	240 kN	0.82	0.0085	0.010	60 kN	170 kN
V1 Bulk	240 kN	0.46	0.0087	0.018		
V2 Linear	110 kN	0.77	0.006	0.007	160 kN	-
V2 Bulk	170 kN	0.8	0.0032	0.004		
V3 Linear	170 kN	0.88	0.013	0.014	150 kN	200 kN
V3 Bulk	135 kN	0.75	0.0058	0.007		
Standard Bricks						
Bulk	200 kN	0.99	0.004	0.004		
Perforated	140 kN	1.11	0.059	0.005		
